# Repurposing Anidulafungin for Alzheimer’s Disease via Fragment-Based Drug Discovery

**DOI:** 10.1021/acschemneuro.4c00150

**Published:** 2024-08-03

**Authors:** Siqi Xie, Yumei Liang, Yang Song, Tingting Li, Jianping Jia

**Affiliations:** † Innovation Center for Neurological Disorders and Department of Neurology, Xuanwu Hospital, Capital Medical University, National Clinical Research Center for Geriatric Diseases, Beijing 100053, P. R. China; ‡ Beijing Key Laboratory of Geriatric Cognitive Disorders, Beijing 100053, P. R. China; § Clinical Center for Neurodegenerative Disease and Memory Impairment, Capital Medical University, Beijing 100053, P. R. China; ∥ Center of Alzheimer’s Disease, Beijing Institute of Brain Disorders, Collaborative Innovation Center for Brain Disorders, Capital Medical University, Beijing 100053, P. R. China; ⊥ Key Laboratory of Neurodegenerative Diseases, Ministry of Education, Beijing 100053, P. R. China

**Keywords:** Alzheimer’s disease, beta amyloid, aggregation inhibitors, anidulafungin, fragment-based drug discovery, drug repurposing

## Abstract

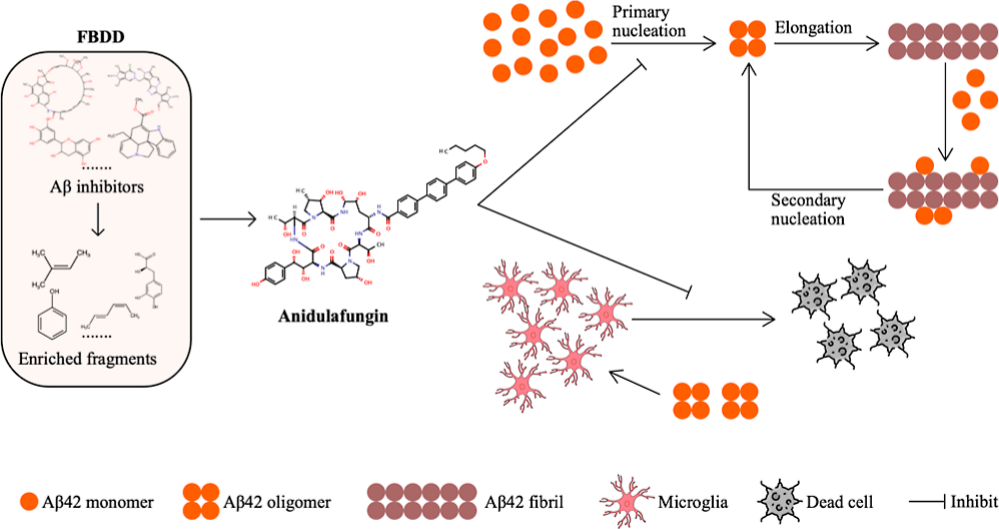

The misfolding and aggregation of beta-amyloid (Aβ) peptides have been implicated as key pathogenic events in the early stages of Alzheimer’s disease (AD). Inhibiting Aβ aggregation represents a potential disease-modifying therapeutic approach to AD treatment. Previous studies have identified various molecules that inhibit Aβ aggregation, some of which share common chemical substructures (fragments) that may be key to their inhibitory activity. Employing fragment-based drug discovery (FBDD) methods may facilitate the identification of these fragments, which can subsequently be used to screen new inhibitors and provide leads for further drug development. In this study, we used an in silico FBDD approach to identify 17 fragment clusters that are significantly enriched among Aβ aggregation inhibitors. These fragments were then used to screen anti-infective agents, a promising drug class for repurposing against amyloid aggregation. This screening process identified 16 anti-infective drugs, 5 of which were chosen for further investigation. Among the 5 candidates, anidulafungin, an antifungal compound, showed high efficacy in inhibiting Aβ aggregation in vitro. Kinetic analysis revealed that anidulafungin selectively blocks the primary nucleation step of Aβ aggregation, substantially delaying Aβ fibril formation. Cell viability assays demonstrated that anidulafungin can reduce the toxicity of oligomeric Aβ on BV2 microglia cells. Molecular docking simulations predicted that anidulafungin interacted with various Aβ species, including monomers, oligomers, and fibrils, potentially explaining its activity against Aβ aggregation and toxicity. This study suggests that anidulafungin is a potential drug to be repurposed for AD, and FBDD is a promising approach for discovering drugs to combat Aβ aggregation.

## 1 Introduction

Alzheimer’s disease (AD) and related dementias have become a major global public health challenge, affecting an estimated 57 million individuals globally in 2019.^1^ The number is expected to rise to 152 million by 2050 due to population growth and population aging. AD accounts for 60–80% of dementia cases.^2^ However, there are currently no treatments that can effectively cure or halt disease progression. Recent immunotherapies targeting β-amyloid (Aβ) protein have demonstrated potential to slow disease progression in trials, but concerns remain regarding potential side effects like amyloid-related imaging abnormalities (ARIAs). Additionally, the high costs of immunotherapies also limit their accessibility.^3,4^ The development of disease-modifying therapeutics for AD remains an unmet need.

Drug repurposing, also known as drug repositioning or reprofiling, offers an alternative strategy to accelerate the development of new AD treatments compared to de novo drug design. Repurposed drugs leverage prior safety and efficacy data established for other indications, expanding the candidate compound pool beyond approved AD medications. They also reduce lengthy timelines and high failure rates that burden traditional clinical trials.^5,6^

A key early event in AD pathogenesis is the misfolding and aggregation of Aβ, followed by tau aggregation, neuronal loss, and ultimately more severe pathological events and clinical manifestations.^7,8^ Blocking Aβ aggregation is thus a promising therapeutic target. Recent findings have revealed several anti-infectives can interfere with aggregation of amyloidogenic proteins like Aβ, tau, and α-synuclein, positioning them as potential repurposing agents against amyloid aggregation in neurodegenerative diseases.^9^

Structure-based drug discovery is an ideal method for developing and screening of drugs to combat Aβ, as its structure plays a key role in aggregation and toxicity.^10,11^ Fragment-based drug discovery (FBDD), a structure-based strategy, could serve as a promising tool to fulfill this goal. FBDD involves screening small, low molecular weight compound fragments to identify those that bind to a target protein, which can then be optimized into potent drug leads.^12^ FBDD can also be employed to analyze small molecule building blocks and identify fragments responsible for the shared properties, like toxicity, across a drug set.^13^

In this study, we employed FBDD to discover fragments potentially underpinning the inhibitory effect on Aβ aggregation of known inhibitors. These fragments were then used to screen for new anti-infective drugs against Aβ aggregation. Furthermore, we validated discovered drugs efficacy and used both kinetic and structural approaches to elucidate the mechanisms of effective drugs.

## 2 Results and Discussion

### 2.1 Identification of Enriched Fragments and Candidate Drugs

#### 2.1.1 Enriched Fragments from Aβ Inhibitors

**Figure 1:**
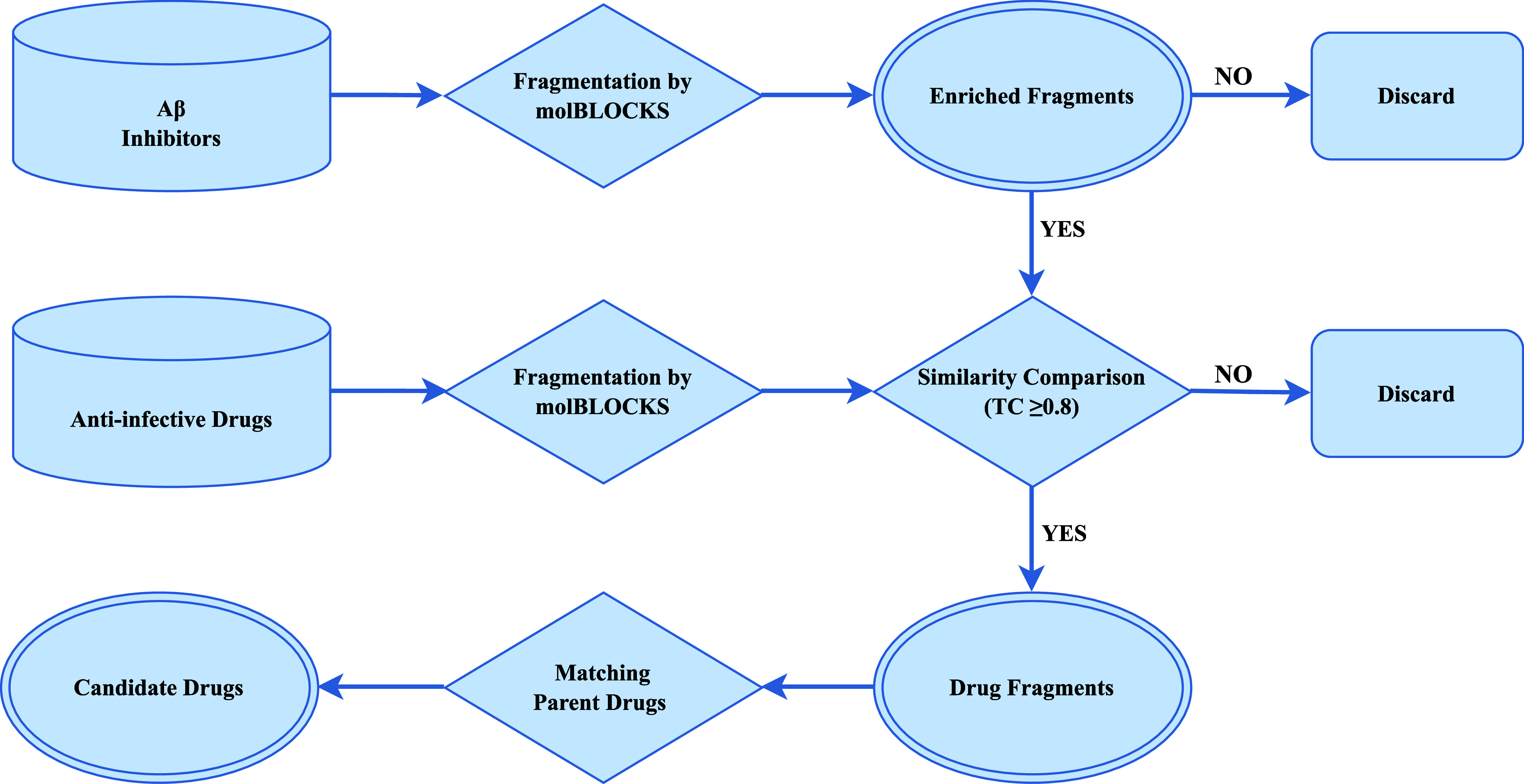
Schematic illustration of the fragment-based drug discovery (FBDD) strategy for identifying novel Aβ42 aggregation inhibitors. This figure outlines the process for identifying anti-infective drugs as potential Aβ42 aggregation inhibitors using a FBDD approach. The process begins with the curation of molecules known to disrupt Aβ aggregation from literature, followed by the retrieval of their structures from PubChem and conversion into simplified molecular-input line-entry system (SMILES) strings. Subsequently, the molBLOCKS software is employed to decompose these structures into fragments, which are then subjected to cluster and enrichment analysis. This analysis aims to identify fragments that occur with significant frequency in Aβ inhibitors beyond chance. These enriched fragments are likely to represent common substructures shared by Aβ inhibitors, conferring Aβ inhibitory function. Next, the enriched fragments are used to screen novel anti-infective drugs from the DrugBank database. These drugs are also fragmented using molBLOCKS. Subsequently, a molecular fingerprint-based comparison is conducted to identify anti-infective drugs containing fragments that are structurally identical or similar (Tanimoto coefficient ≥0.8) to the enriched fragments. Finally, the parent drugs containing these fragments are identified as promising candidates with potential Aβ42 aggregation inhibition activity.

Figure 1 illustrates the process used to identify enriched fragment subsets within Aβ inhibitors and subsequently screen for potential anti-infective agents.

An initial library of 148 molecules known to disrupt Aβ aggregation was compiled from published literature. These molecules were fragmented, clustered, and subjected to enrichment analysis using the molBLOCKS software. A total of 17 molecular fragments exhibited significant enrichment with a false discovery rate (FDR) of less than 0.01, indicating that these fragments appear in Aβ inhibitors with a significantly higher frequency than by chance. These fragments may thus be related to the inhibitory effect of those Aβ inhibitors. Table 1 outlines the structures and frequencies of these enriched fragments, which fall into three major structural categories: aromatic rings, phenolic rings, and alkenyl chains.

**Table 1:**
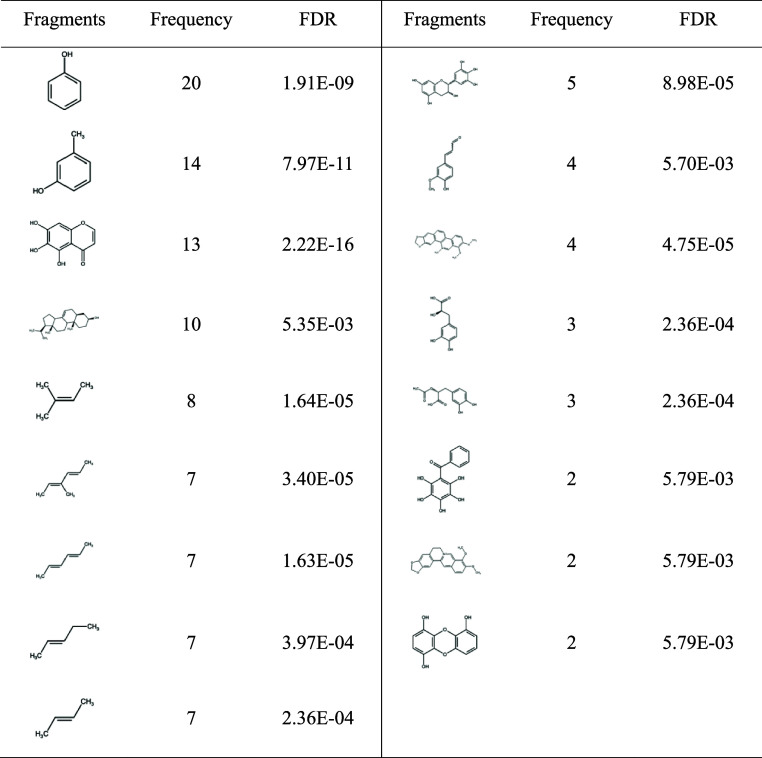
Enriched Fragments from Aβ Inhibitors

#### 2.1.2 Anti-Infective Drugs with Enriched Fragments

A set of 463 anti-infective drugs, classified according to their Anatomical Therapeutic Chemical (ATC) codes, was chosen for analysis. After excluding 9 entries lacking structural data, the remaining 454 drugs were fragmented using molBLOCKS. Comparison of the structural similarity of these fragments against the 17 enriched fragments from Aβ inhibitors (applying a Tanimoto coefficient threshold of ≥0.8) led to the identification of 16 anti-infective drugs containing enriched fragments in their structures, making them promising candidates as Aβ inhibitors. This candidate list comprises the following agents: antifungals (anidulafungin, micafungin, ketoconazole, itraconazole, oteseconazole, posaconazole, pecilocin), antibiotics (phenoxymethylpenicillin, pheneticillin, carfecillin, propicillin, penimepicycline), antivirals (lopinavir, pleconaril), an antituberculosis agent (delamanid), and an antiseptic (resorcinol). Table 2 provides details on these candidate drugs.

**Table 2:**
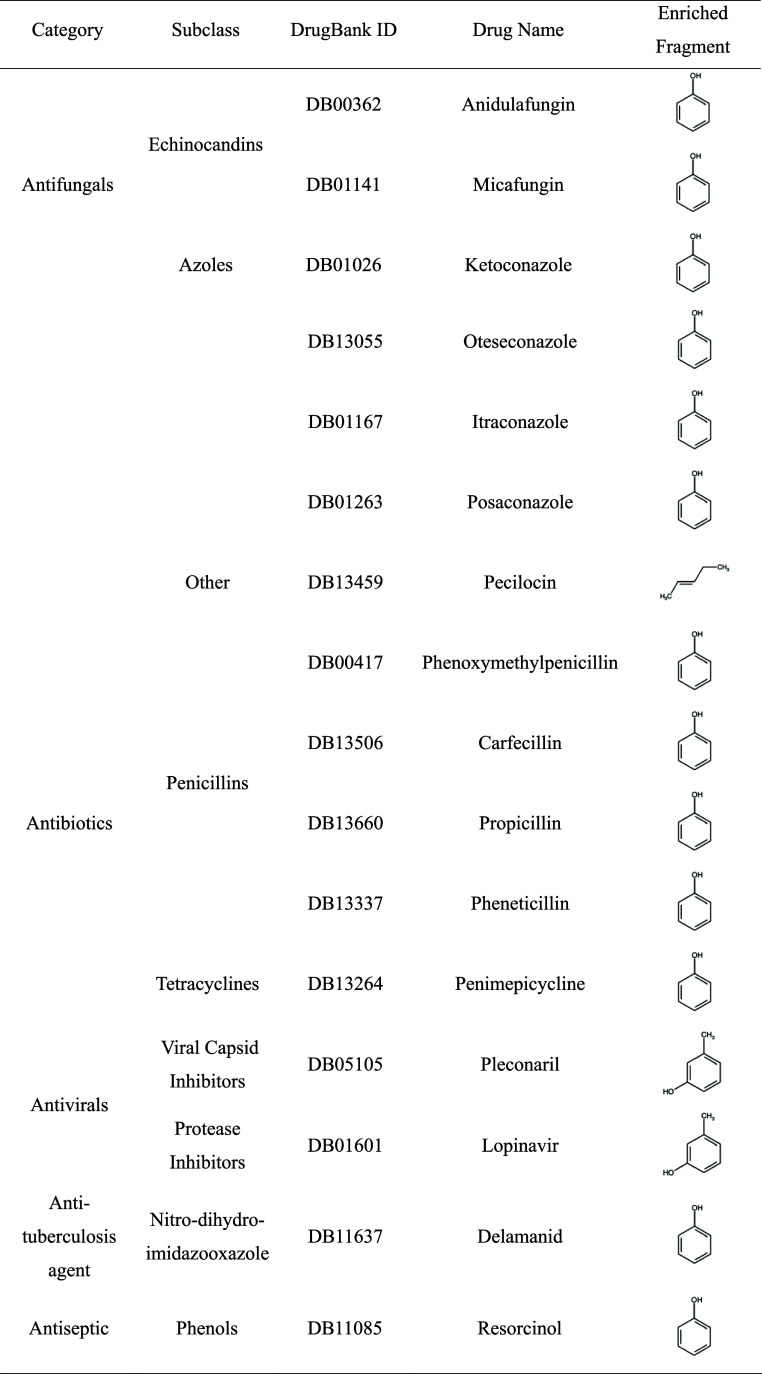
Candidate Drugs with Enriched Fragments from Aβ Inhibitors

### 2.2 Effects of Candidate Drugs on Aβ Aggregation

After a literature review and exclusion of compounds with known Aβ aggregation properties, we selected five candidate drugs for further investigation: anidulafungin, itraconazole, oteseconazole, delamanid, and pleconaril.

To assess the impact of these drugs on Aβ42 aggregation, we conducted in vitro aggregation assays using 5 μM Aβ42 with varying drug concentrations. The ability to disrupt Aβ42 aggregation was determined by observing aggregation kinetics, which characteristically exhibit sigmoidal growth with distinct lag, growth, and plateau phases.^14^

**Figure 2:**
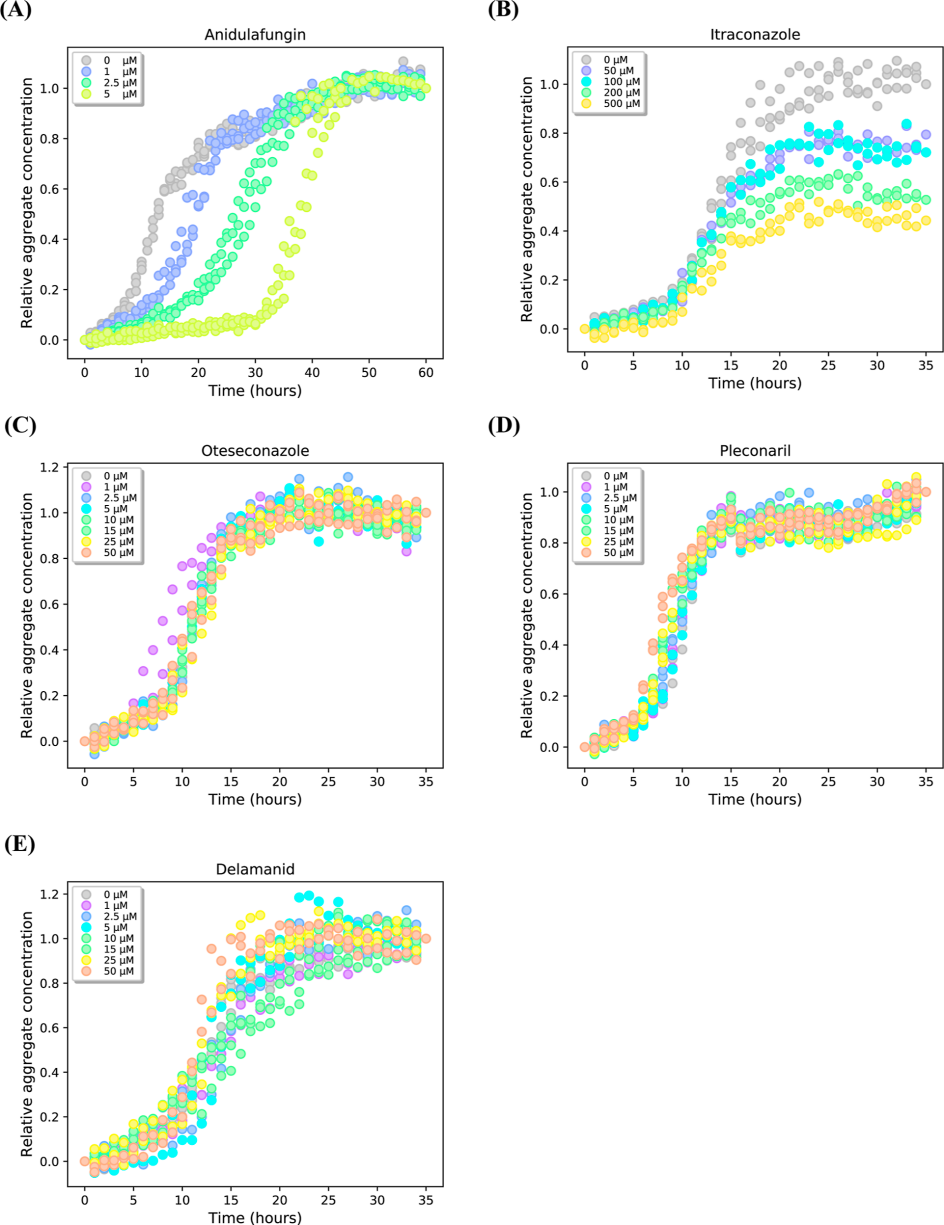
Effects of candidate drugs on in vitro Aβ42 aggregation. This figure shows aggregation kinetics of 5 μM Aβ42 in the presence and absence of five candidate drugs at varying concentrations. (A) Anidulafungin exhibited dose-dependent inhibition of Aβ42 aggregation, with delayed onset observed at a 1:5 drug-to-Aβ42 ratio and significant suppression at a 1:1 ratio. (B) Itraconazole moderately reduced Aβ42 fibril formation, achieving a 20% reduction at a 10:1 drug-to-Aβ42 ratio and a 50% reduction at a 100:1 ratio. (C–E) Oteseconazole, pleconaril, and delamanid failed to inhibit 5 μM Aβ42 aggregation, even at a 10:1 drug-to-Aβ42 concentration ratio.

Anidulafungin demonstrated the most potent inhibitory effect on Aβ42 aggregation. At a 1:5 drug-to-Aβ42 ratio, aggregation was delayed, and a 1:1 ratio significantly extended the lag phase (Figure 2A). Itraconazole dose-dependently reduced total Aβ42 fibril formation, evident from decreased Thioflavin T (ThT) fluorescence during the plateau phase. However, this effect required a 10:1 drug-to-Aβ42 ratio, and a 50% reduction in fibril formation was achieved only at a 100:1 ratio(Figure 2B). Oteseconazole, delamanid, and pleconaril failed to impact the aggregation of 5 μM Aβ42 at concentrations up to 50 μM (Figure 2C–E). The lack of observed effects suggest that these three drugs may not impede Aβ42 aggregation. Based on these results, anidulafungin emerged as the most promising for further investigation, given its ability to significantly alter the aggregation kinetics of Aβ42.

### 2.3 Effects of Anidulafungin on Microscopic Steps in Aβ42 Aggregation

Aβ42 aggregation involves primary nucleation, secondary nucleation, and fibril elongation. Primary nucleation is the rate-limiting step where Aβ42 monomers form unstable nuclei that seed further aggregation. These nuclei mature into oligomers, protofibrils, and ultimately fibrils (elongation phase). Once a sufficient fibril concentration is reached, secondary nucleation becomes the dominant mechanism, where fibrils catalyze further oligomers formation and create a positive feedback loop that accelerates aggregation.^15^

**Figure 3:**
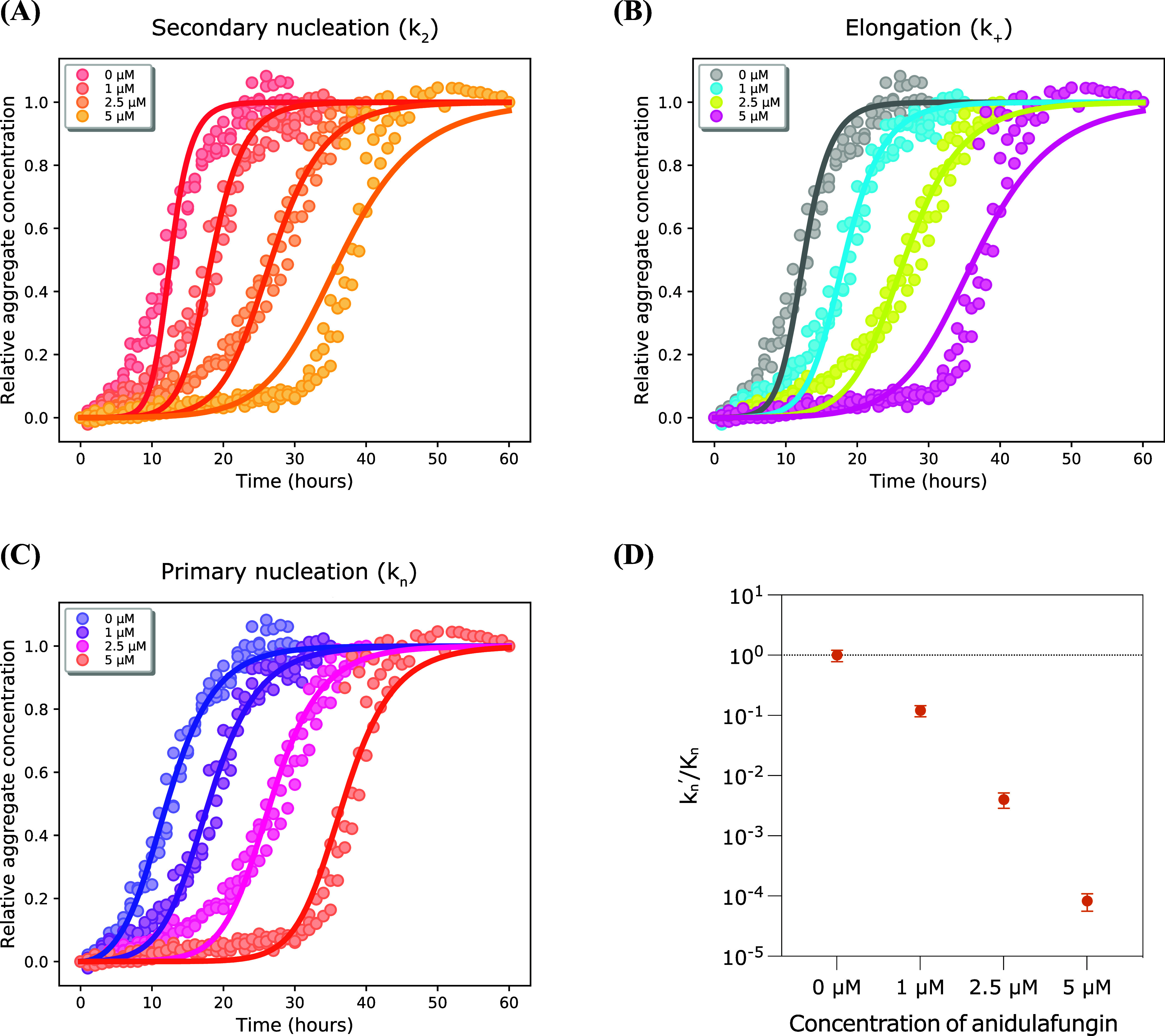
Anidulafungin targets the primary nucleation step of Aβ42 aggregation. (A–C) Global kinetic analysis of 5 μM Aβ42 aggregation with varying anidulafungin concentrations (1, 2.5 and 5 μM). Solid lines depict model-predicted reaction profiles with selective inhibition of secondary nucleation (A), elongation (B), or primary nucleation (C). Experimental data closely match the model only when primary nucleation is inhibited. (D) Anidulafungin inhibits primary nucleation of 5 μM Aβ42 in a dose-dependent manner. The rate constant of primary nucleation (*k*_*n*_) decreases with increasing anidulafungin concentrations. *k*_*n*_′ represents the rate constant in the presence of anidulafungin at varying concentrations, whereas *K*_*n*_ denotes the rate constant for 5 μM Aβ42 alone.

We used the AmyloFit platform to perform a global kinetic analysis of 5 μM Aβ42 aggregation in the presence of varying anidulafungin concentrations. This analysis yields rate constants for each microscopic step (*k*_*n*_: primary nucleation; *k*_+_: elongation; *k*_2_: secondary nucleation), clarifying how anidulafungin influences Aβ42 aggregation. Employing the “secondary nucleation dominated” model, we fit one rate constant at a time to precisely identify the affected step. Aggregation profiles of 5 μM Aβ42 with 1–5 μM anidulafungin closely matched the model’s predictions only when selectively fitting the primary nucleation rate constant (*k*_*n*_). Conversely, experimental data showed poor agreement when fitting secondary nucleation (*k*_2_) or elongation (*k*_+_) rate constants (Figure 3A–C). Further analysis revealed that anidulafungin reduced the rate of primary nucleation in a concentration-dependent manner (Figure 3D).

### 2.4 Dot Blot and AFM Analysis Confirm Anidulafungin-Mediated Delay in Aβ42 Fibril Formation

**Figure 4:**
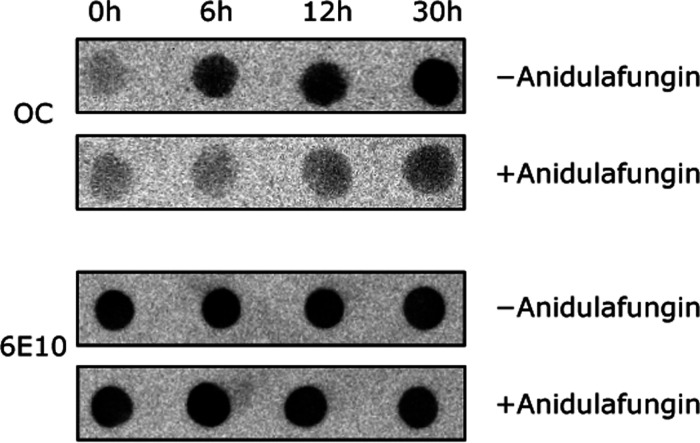
Dot blot analysis reveals delayed Aβ42 fibril formation in the presence of anidulafungin. Time course of the formation of 5 μM Aβ42 fibrils as assessed by antibody binding. The fibril-specific OC antibody probes only fibrillar structures (upper panel). Without anidulafungin, detectable fibril formation occurred by 6 h, with significantly increased signal intensity observed at 30 h. In the presence of 5 μM anidulafungin, fibril formation was significantly delayed, resulting in consistently weaker OC antibody signals throughout the incubation period. In contrast, the sequence-specific 6E10 antibody detects all Aβ42 species. The quantity of total Aβ42 detected by the 6E10 antibody (lower panel) remained unchanged throughout the entire time course, both with and without anidulafungin.

Dot blot analysis and Atomic Force Microscopy (AFM) imaging support the kinetic analysis. We probed the quantities of Aβ42 at different time points during aggregation using either the fibril-specific OC or the sequence-specific 6E10 primary antibodies. The fibril-specific OC antibody showed that 5 μM Aβ42 alone formed detectable fibrils by 6 h, with a robust OC signal at 30 h. In the presence of 5 μM anidulafungin, the OC signal was significantly reduced throughout the incubation period, indicating lower fibril content (Figure 4). In contrast, since the 6E10 antibody is sequence-specific, it binds to all types of Aβ42 species. The 6E10 antibody indicated the presence of similar quantities of Aβ42 at different time points during the aggregation reaction, both with and without anidulafungin.

**Figure 5:**
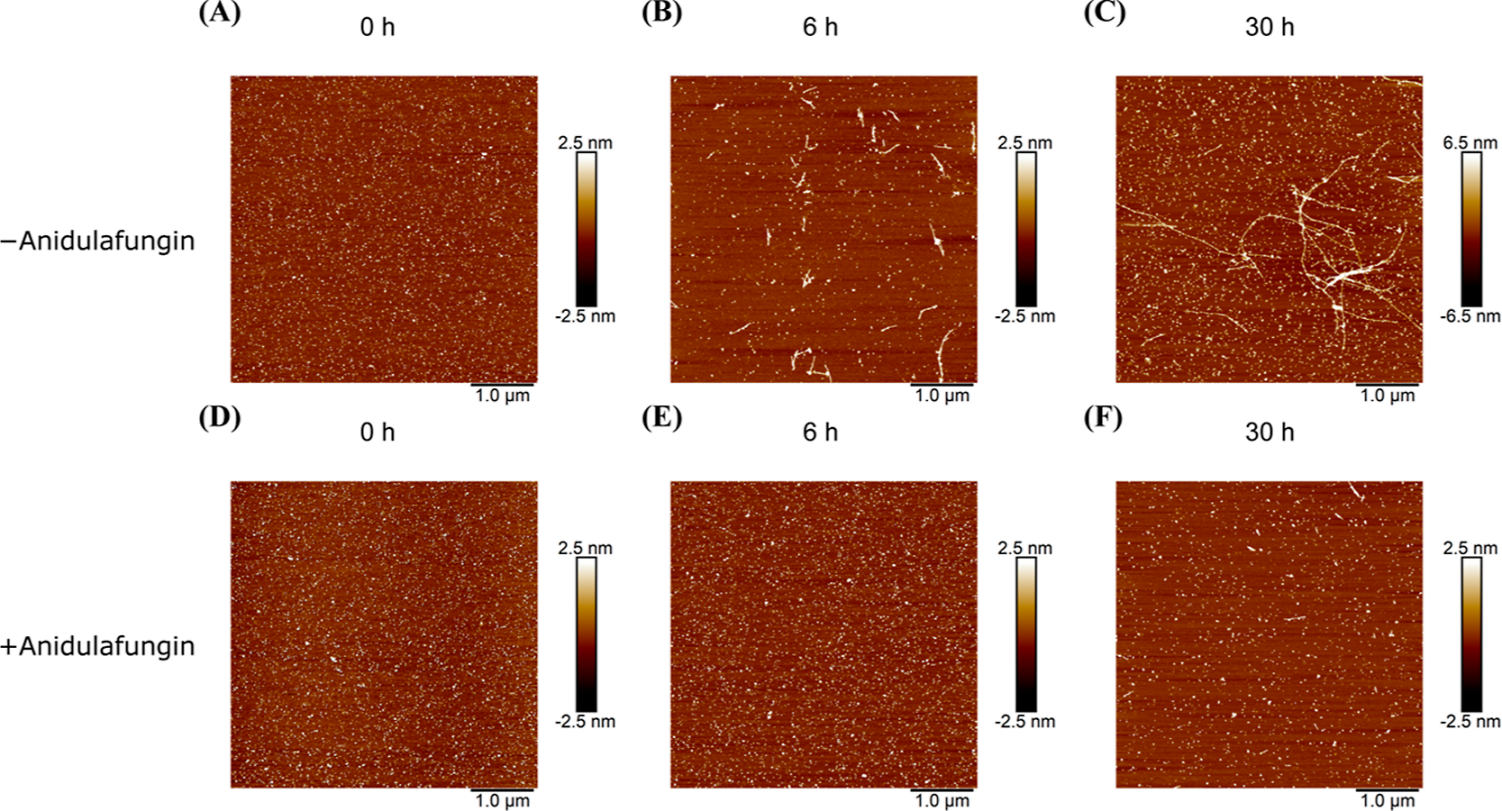
Anidulafungin delays the formation of Aβ42 fibrils. AFM images of Aβ42 species in the absence and presence of anidulafungin at a 1:1 ratio to Aβ42. In the absence of anidulafungin, fibril formation is evident at 6 h (B), and mature fibrils are present by 30 h (C). Conversely, anidulafungin-treated samples show no fibrils at 6 h (D) and only limited formation of short aggregates by 30 h (F). Scale bar = 1.0 μM.

AFM analysis also confirmed delayed fibril formation in the presence of anidulafungin (Figure 5). Without anidulafungin, initial 5 μM Aβ42 monomers rapidly aggregated, forming extensive short structures by 6 h and mature fibrils by 30 h. In the presence of 5 μM anidulafungin, Aβ42 species at 6 h remained primarily monomeric, with only larger oligomers and short fibrils evident at 30 h. These combined results clearly demonstrate that 5 μM anidulafungin significantly delays Aβ42 fibril formation.

### 2.5 Anidulafungin Mitigates Aβ Oligomers-Induced Cytotoxicity in BV2 Cells

**Figure 6:**
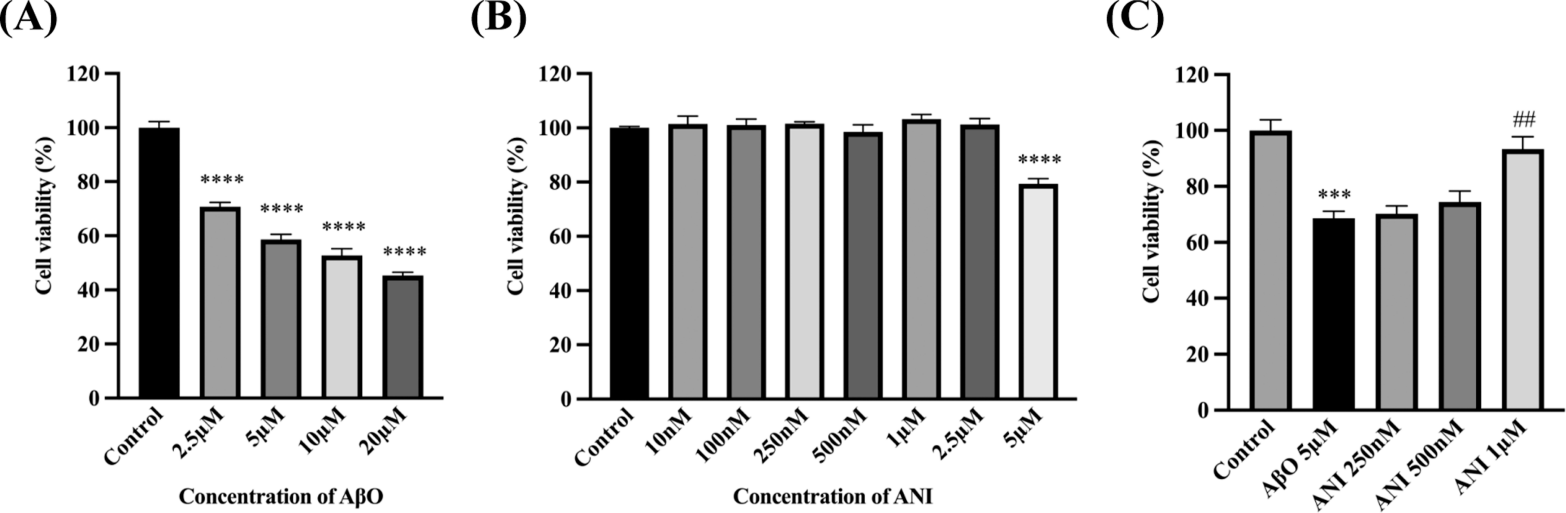
Anidulafungin protects BV2 cells against AβO-induced cytotoxicity. (A) AβO (2.5–20 μM) dose-dependently reduced BV2 cell viability. Based on this, we selected 5 μM AβO treatment to establish cytotoxicity model. (B) Anidulafungin was nontoxic to BV2 cells within the tested range (10 nM–2.5 μM). (C) Co-treatment with 1 μM anidulafungin significantly mitigated 5 μM AβO cytotoxicity, restoring cell viability to 93.30% compared to 68.66% with AβO alone (*P* < 0.01). Error bars represent mean ± SEM (*n* = 4). **** means *P* < 0.0001 when compared to the control group, *** means *P* < 0.001 when compared to the control group, ## means *P* < 0.01 when compared to the 5 μM AβO treated group. ANI: anidulafungin, AβO: Aβ42 oligomers.

Cell Counting Kit-8 (CCK-8) assays were used to assess Aβ oligomer (AβO)-induced cytotoxicity in BV2 cells and the protective effects of anidulafungin against AβO. First, BV2 cells were subjected to 2.5–20 μM AβO, and a dose-dependent cytotoxicity was observed. We then used 5 μM AβO to establish the AβO cytotoxicity model. Anidulafungin was added to BV2 cells and was shown to be nontoxic within the range of 10 nM to 1 μM. Subsequently, different concentrations of anidulafungin were added to BV2 cells combined with 5 μM AβO. We found that 1 μM anidulafungin significantly attenuated 5 μM AβO-induced cytotoxicity in BV2 cells, restoring cell viability to 93.30% compared to 68.66% with AβO alone (*P* < 0.01, Figure 6).

### 2.6 Molecular Docking Suggests Interactions between Anidulafungin and Various Aβ42 Structures

The Aβ42 peptide’s sequence (DAEFRHDSGY^10^EVHHQKLVFF^20^AEDVGSNKGA^30^IIGLMVGGVV^40^IA) contains four distinct domains: a hydrophilic N-terminus (1DAEFRHDSGYEVHHQK16), a central hydrophobic core (17LVFFA21), a central hydrophilic region (22EDVGSNKG29), and a hydrophobic C-terminus (30AIIGLMVGGVVIA42).^16^

**Figure 7:**
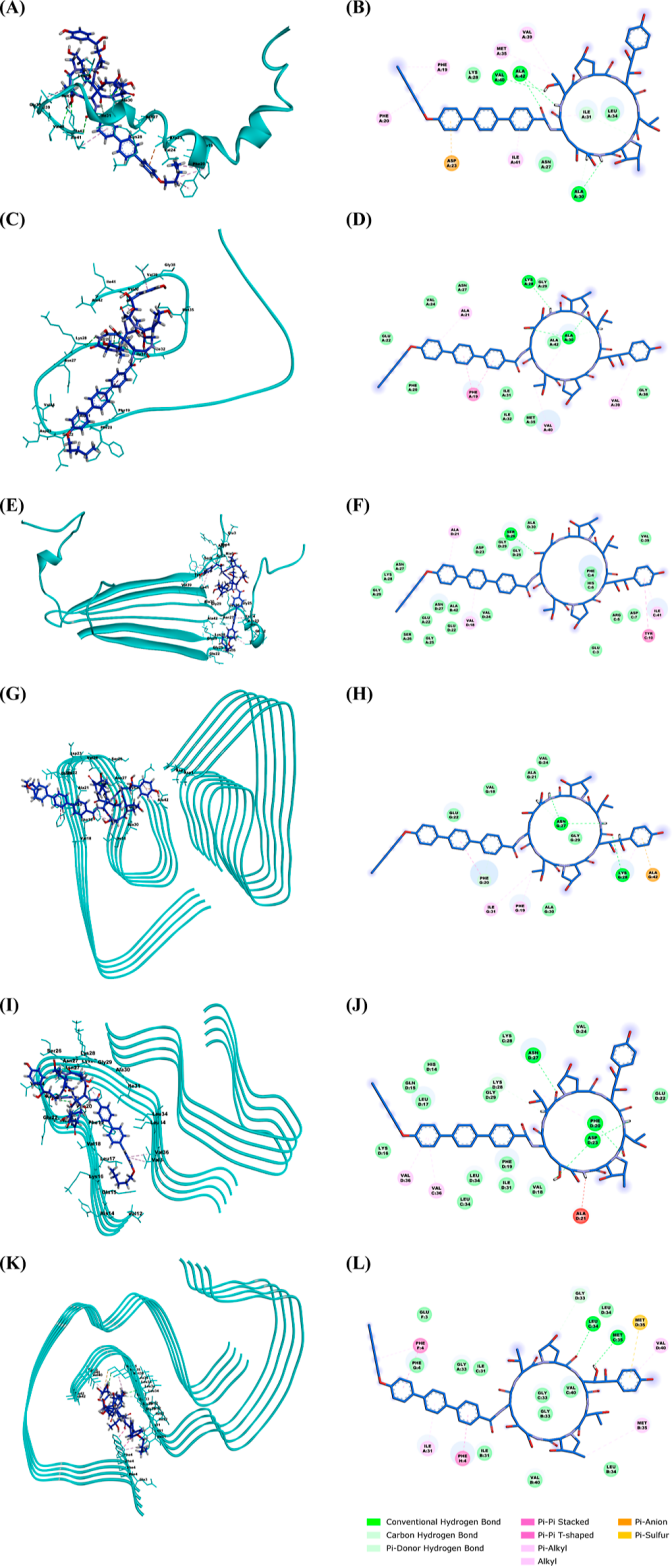
Molecular docking simulations predict anidulafungin binding to diverse Aβ42 conformations. Molecular docking was used to predict the potential interactions of anidulafungin with various Aβ42 structures. Both three-dimensional (left) and two-dimensional (right) representations of the predicted binding modes are shown for: (A,B) α-helical monomer (1Z0Q); (C,D) β-sheet monomer (5OQV); (E,F) tetramer (6RHY); (G,H) LS-shaped fibril (5OQV); (I,J) ν-shaped fibril (8EZD); (K,L) υ-shaped fibril (8EZE). Simulations predict favorable binding of anidulafungin to all tested conformations, suggesting a potential mechanism for its observed inhibition of Aβ42 aggregation and toxicity.

To investigate the potential molecular mechanism by which anidulafungin inhibits Aβ42 aggregation, we performed molecular docking simulations with a range of Aβ42 conformations. These included monomers (α-helical and β-sheet), tetramers, and LS/ν/υ-shaped fibrils (Figure 7). The simulations revealed that anidulafungin binds to the α-helical Aβ42 monomer with a binding energy of −6.471 kcal/mol, through 6 hydrogen bond interactions, 7 hydrophobic interactions, and 1 electrostatic interaction. For the β-sheet Aβ42 monomer, anidulafungin binds with a binding energy of −7.148 kcal/mol throught 4 hydrogen bond interactions and 6 hydrophobic interactions. Anidulafungin’s interaction with Aβ42 tetramers occurs through 1 hydrogen bond interaction and 4 hydrophobic interactions, with a binding energy of −8.159 kcal/mol. For LS-shaped Aβ42 fibrils, anidulafungin binds with a binding energy of −7.557 kcal/mol, through 7 hydrogen bond interactions, 5 hydrophobic interactions, and 1 electrostatic interaction. In the case of ν-shaped Aβ42 fibrils, anidulafungin binds with a binding energy of −7.267 kcal/mol, through 5 hydrogen bond interactions and 3 hydrophobic interactions. For υ-shaped Aβ42 fibrils, anidulafungin binds with a binding energy of −8.629 kcal/mol, through 3 hydrogen bond interactions, 6 hydrophobic interactions, and 1 electrostatic interaction. Detailed information on these binding interactions is presented in Table 3.

**Table 3:** Binding Energies and Interaction Information of Anidulafungin with Various Aβ42 Structures^a^

receptors	binding energy (kcal/mol)	interacting residues		
		H-bond interaction	hydrophobic interaction	electrostatic interaction
α-helical Aβ42 monomer (1Z0Q)	–6.471	Ala30	Phe19 (π-alkyl)	Asp23 (π-anion)
		Ile31	Phe20 (π-alkyl)	
		Vla40	Ile31 (π-alkyl)	
		Ala42	Met35 (alkyl)	
			Val39 (alkyl)	
			Ile41 (π-alkyl)	
β-sheet Aβ42 monomer (5OQV)	–7.148	Lys28	Phe19 (π–π stacked)	
		Ala30	Ala21 (π-alkyl)	
			Ala30 (alkyl)	
			Val39 (π-alkyl)	
			Vla40 (alkyl)	
Aβ42 tetramers (6RHY)	–8.159	Ser26^D^	Tyr10^C^ (π–π T-shaped)	
			Val18^D^ (π-alkyl)	
			Ala21^D^ (π-alkyl)	
			Ile41^C^ (π-alkyl)	
LS-shaped Aβ42 fibrils (5OQV)	–7.557	Phe20^G^	Phe19^G^ (π-alkyl)	Ala42^G^ (π-anion)
		Asn27^G^ Lys28^G^	Phe20^G^ (π–π T-shaped)	
			Lys28^G^ (π-alkyl)	
			Ile31^G^ (alkyl)	
			Ala42^G^ (π-alkyl)	
ν-shaped Aβ42 fibrils (8EZD)	–7.267	Phe20^D^	Phe20^D^ (π-alkyl)	
		Asp23^D^	Vla36^C^ (π-alkyl)	
		Asn27^D^	Vla36^D^ (π-alkyl)	
		Lys28^D^		
υ-shaped Aβ42 fibrils (8EZE)	–8.629	Gly33^D^ Leu34^C^	Phe4^F^ (π–π T-shaped, π-alkyl)	Met35^D^ (π-sulfur)
		Met35^C^	Phe4^H^ (π–π stacked)	
			Ile31^A^ (π-alkyl)	
			Met35^B^ (alkyl)	
			Vla40^D^ (π-alkyl)	

a The lowercase letters at the upper right of the amino acid residue denote the specific peptide chain in which the residue is located.

### 2.7 Discussion

Structure-based design has proven effective in developing protein, peptide, and small molecule inhibitors against amyloid aggregation.^17−21^ Another notable example is the FBDD-guided repurposing of the anticancer drug bexarotene as an Aβ42 aggregation inhibitor.^22^ However, systematic strategies to identify potentially functional fragments within inhibitors for Aβ42 aggregation have been lacking. This study addressed this gap by employing molBLOCKS software for fragment discovery, with its specific rules for compound decomposition and functional fragment enrichment. Our analysis revealed an enrichment of three structural types within Aβ42 aggregation inhibitors: aromatic, phenolic, and alkenyl fragments.

These findings align with previous studies demonstrating the role of specific structures in disrupting Aβ aggregation. Aromatic ring structures have been reported to disrupt the ordered β-sheet stacking and interfere with Aβ42 aggregation through π–π interactions,^23,24^ which are critical for amyloid self-assembly.^25,26^ Phenolic groups, found in natural products like polyphenols and flavonoids, also exert inhibitory effects on Aβ aggregation.^27−30^ These phenolic moieties interact with aromatic residues in Aβ through π–π stacking or hydrophobic forces and can disrupt Aβ aggregates.^31−33^ Furthermore, the polyene backbone formed by multiple alkenyl groups is a feature of carotenoids, known Aβ aggregation inhibitors.^34,35^ These polyene structures impede Aβ fibril formation through hydrophobic interactions, potentially blocking peptide stacking.^34,36,37^

Screening a library of anti-infective drugs containing the enriched fragments yielded 16 promising candidates for Aβ42 aggregation inhibition. Notably, the majority were antifungals, including four azoles, two echinocandins, and one natural product. Other classes identified were penicillin and tetracycline antibiotics, antivirals, an antituberculosis agent, and an antiseptic. Some of these drug classes have been reported to interfere with Aβ aggregation. For instance, azole compounds have demonstrated inhibitory effects against several Aβ aggregation pathways,^38−41^ but the specific azole antifungals identified in our study require further investigation. Caspofungin, an echinocandin, has recently been repurposed against Aβ aggregation. It reduces Aβ′s β-sheet formation tendency, prolongs the aggregation lag phase, and promotes amorphous aggregates formation.^42^ Penicillin antibiotics, such as benzylpenicillin, can bind Aβ covalently and modulate aggregation through β-lactam ring interactions.^43^ Tetracyclines, well-known for their antiamyloidogenic effects,^44^ include compounds like doxycycline and minocycline, which inhibit Aβ aggregation pathways, generate nontoxic amorphous aggregates, and disassemble mature fibrils.^45−47^

For experimental validation, we selected five drugs: anidulafungin, itraconazole, oteseconazole, pleconaril, and delamanid. Notably, the antifungal anidulafungin emerged as the most potent inhibitor of Aβ42 aggregation in vitro. Itraconazole also exhibited moderate inhibitory activity, albeit at higher concentrations. Furthermore, kinetic analyses revealed that anidulafungin selectively targets primary nucleation, the earliest stage of Aβ42 aggregation, and significantly reduces nucleation rates. When anidulafungin was added to Aβ42 at a 1:2 concentration ratio, it reduced the primary nucleation rate constant (*k*_*n*_) by more than 2 orders of magnitude, decreasing it to 0.004 compared to its value in the absence of anidulafungin. This potency is comparable to that of the antibody DesAb_18–25_, which specifically targets primary nucleation and decreases *k*_*n*_ by 2 orders of magnitude (to 0.0054) at a 1:2 antibody-to-Aβ42 ratio.^48^ Moreover, anidulafungin’s potency surpasses that of bexarotene, another inhibitor, which decreases *k*_*n*_ by 1 order of magnitude (to 0.1) at a 1:1 drug-to-Aβ42 ratio.^22^ These findings suggest anidulafungin’s potential in AD prevention by slowing the formation of primary nuclei, which are crucial for exponential fibril growth.^15,49^

As the most potent inhibitor of Aβ42 aggregation, we further analyzed anidulafungin’s impact on Aβ42 aggregation and Aβ-induced cytotoxicity in vitro. AFM enabled high-resolution visualization of Aβ42 species formed during aggregation, confirming that anidulafungin delays mature fibril formation. This finding was supported by dot blot analysis with a fibril-specific antibody. Moreover, 1 μM anidulafungin attenuated the cytotoxicity of 5 μM Aβ42 oligomers on BV2 microglia.

Molecular docking simulations provided insights into the potential mechanisms by which anidulafungin disrupts Aβ42 aggregation. Anidulafungin was predicted to interact with the α-helical Aβ42 monomer via hydrogen bonds, hydrophobic interactions, and electrostatic interactions, predominantly at the hydrophobic C-terminal region and central hydrophobic core–both crucial sites for aggregation. The C-terminal residues Ile41 and Ala42 confer marked rigidity and stabilize turn conformation that critical for oligomerization.^50,51^ Meanwhile, the central hydrophobic core represents a key amyloidogenic sequence and the site of nucleation or self-recognition.^52^ By interacting with these residues, anidulafungin could block initial nucleus formation by disrupting monomer–monomer interactions and β-sheet transformation. Anidulafungin also displayed hydrogen bonds and hydrophobic interactions with β-sheet structured Aβ42 monomers at the same critical C-terminal and hydrophobic core regions, likely hindering mature nucleus formation to impede aggregation. The enriched fragment in anidulafungin is a phenol group located in its macrocycle, which predominantly interacts with the C-terminal region of Aβ42. Previous studies have demonstrated that these structures create a hydrophobic cavity, which serves as a crucial active site for Aβ inhibition.^53^ Since this macrocyclic structure is absent in the other four molecules tested in the in vitro aggregation assay, it might explain the unique inhibitory activity of anidulafungin. Additionally, interactions were predicted between anidulafungin and toxic Aβ42 species like tetramers and fibrils, thereby reducing their toxicity.

Overall, this study suggests that anidulafungin, an antifungal drug, demonstrates promising inhibitory activity against Aβ42 aggregation, opening up the possibility for its repurposing in AD. We also highlights the potential of structure-based drug design strategies, like FBDD, for identifying new inhibitors of Aβ42 aggregation. This approach could be extended to screen and design inhibitors against other amyloidogenic proteins, such as tau and α-synuclein. Given the shared structural and chemical features among these proteins, there’s a promising opportunity to develop multitarget inhibitors capable of simultaneously disrupting the aggregation of different amyloid species.

## 3 Materials and Methods

### 3.1 Discovery of Enriched Molecule Fragments in Aβ Inhibitors

To compile a library of previously reported Aβ inhibitors, we searched the PubMed database (2013–2023) using the query terms “beta amyloid” or “Aβ” in combination with terms related to aggregation and inhibition (“aggregate”, “assemble”, “inhibit”, “disrupt”). This search yielded 148 compounds. We downloaded structure data files (SDF) of these compounds from PubChem and converted them to SMILES using Open Babel.^54^

We used the molBLOCKS for fragment discovery.^55^ molBLOCKS is a software suite designed for FBDD. It consists of two main programs: “fragment” and “analysis”. The “fragment” program decomposes molecules into chemically meaningful fragments, while the “analysis” program clusters similar fragments and identifies enriched substructures that occur more frequently than expected by chance against a background fragment set using a hypergeometric distribution.

In this study, inhibitor molecules were decomposed into fragments using the “fragment” program by applying the RECAP rule for fragmentation.^56^ The resulting fragments were clustered based on a Tanimoto coefficient threshold of ≥0.8. We performed enrichment analysis against a background set of 5000 randomly selected PubChem molecules with matched structural complexity. Fragments were considered enriched at a FDR corrected *P*-value <0.01.

### 3.2 Identification of Anti-Infective Drugs with Enriched Fragments

Anti-infective drugs were identified through the DrugBank database utilizing the ATC classification system.^57^ This system organizes drugs according to their targeted organs or systems and therapeutic actions. For this study, drugs classified within anti-infective categories (A07AA, C05AB, D01, D06A, D06BB, G01AA, J01, J02, J04, J05, L01D, and S01A) were selected as candidates and subsequently fragmented using molBLOCKS. Fragments derived from this drug data set were then compared against the enriched fragments identified from Aβ inhibitors. Drugs containing at least one enriched fragment or a structurally similar fragment (Tanimoto coefficient ≥0.8) were selected for further analysis. Figure 1 provides a schematic overview of the strategy employed for identifying enriched fragments from Aβ aggregation inhibitors and screening for potential anti-infective drug candidates.

### 3.3 In Vitro Aβ42 Aggregation Assay

Synthetic human Aβ42 peptide (Abcam) was dissolved in 1,1,1,3,3,3-hexafluoroisopropanol (HFIP) to a 1 mg/mL concentration and maintained at room temperature until clear. The solution was sonicated in a water bath for 5 min, placed on ice, and aliquoted into low-bind tubes (Corning). After overnight evaporation of HFIP in a fume hood, the peptide film was stored at −80 °C. For experiments, the peptide film was reconstituted in 60 mM NaOH to 200 μM, incubated at room temperature for 20 min, and sonicated in an ice-water bath for 15 min. This solution was then diluted to 20 μM in cold buffer (20 mM sodium phosphate, 200 μM EDTA, and 1 mM NaN3).

For aggregation assays, the 20 μM Aβ solution was combined with ThT in the same buffer to a final volume of 200 μL in a 96-well plate. Final Aβ42 and ThT concentrations were 5 and 20 μM, respectively. Aggregation was induced by incubation at 37 °C, and fluorescence (excitation: 440 nm, emission: 480 nm) was measured at 6 min intervals.

For drug-treatment experiments, drugs were dissolved in DMSO to create stock solutions. These were diluted in 20 mM sodium phosphate buffer to desired concentrations, maintaining a final DMSO concentration below 1%. The drug solutions were then added to the Aβ/ThT mixture to achieve a final volume of 200 μL.

### 3.4 Kinetics Analysis of Aβ42 Aggregation

The kinetic analysis of Aβ42 aggregation was conducted using AmyloFit, an online platform designed for the global analysis of protein aggregation kinetics.^58^ The following integrated rate law is used to describe the generation of total fibril mass^15^
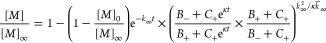
where the definitions of the parameters are
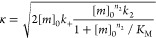

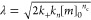

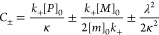

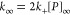

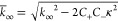

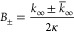
[*m*]_0_ is the initial monomer concentration; [*P*]_0_ is the initial fibril number concentration; [*P*]_∞_ is the fibril number concentration at equilibrium, when the reaction has reached completion; [*P*]_0_ and [*P*]_∞_ are the initial and equilibrium fibril number concentrations, respectively; [*M*]_0_ and [*M*]_∞_ are the initial and equilibrium fibril mass concentrations; *k*_*n*_, *k*_2_, *k*_+_ are the rate constants for primary nucleation, secondary nucleation, and elongation, respectively; *K*_M_ is the saturation constant for secondary nucleation; *n*_c_ and *n*_2_ are the reaction orders of primary and secondary nucleation, respectively.

In this study, the model “secondary nucleation dominated” was used, with reaction orders *n*_c_ and *n*_2_ both set to 2 based on previous studies of Aβ42 aggregation.^15,59^ The initial seed concentration [*M*]_0_ was set to 0. To investigate the specific effects of inhibitors on the aggregation process, three separate fits were performed. Each fit allowed one rate constant (*k*_*n*_, *k*_2_ or *k*_+_) to vary as an independent parameter, while the remaining two were set “global fit” across the data set. This means that the two nonvarying rate constants were assigned identical values for all groups. This approach allowed us to analyze deviations from the global parameters, revealing whether selective perturbation of primary nucleation, secondary nucleation, or elongation could best explain the observed inhibition patterns. Ultimately, this strategy helps identify which microscopic steps in the aggregation process are most likely targeted by the inhibitors.

### 3.5 Dot Blot Analysis

Samples from the aggregation assay were collected at different time points and stored at −80 °C. Before analysis, samples were thawed on ice and sonicated in an ice-water bath for 2 min for homogenization. The polyvinylidene fluoride (PVDF) membrane (Millipore) was activated with 100% ethanol for 1 min, washed with distilled water for 2 min, and equilibrated in Tris-buffered saline with Tween 20 (TBS-T; 20 mM Tris, 150 mM NaCl, 0.05% Tween 20, pH 7.5).

The membrane was placed on TBS-T soaked filter paper, and 2 μL of each sample was spotted onto the membrane within a premarked grid. After air-drying at room temperature for 1 h, the membrane was blocked with blocking buffer (NCM Biotech) for 20 min. Following a 5 min TBS-T rinse, the membrane was incubated overnight at 4 °C with either the antiamyloid fibrils OC antibody (Sigma-Aldrich, 1:20,000 dilution) or the anti-β-amyloid 1–16 antibody (6E10, BioLegend, 1:1000 dilution).

Three 10 min TBS-T rinses were performed before incubation with an HRP-conjugated goat antirabbit IgG secondary antibody (ImmunoWay, 1:5000 dilution) or goat antimouse IgG/HRP antibody (Solarbio, 1:5000 dilution). The membrane was rinsed again (three 10 min TBS-T washes) and visualized using an enhanced chemiluminescence kit (NCM Biotech).

### 3.6 Atomic Force Microscopy

AFM was used to characterize the morphology of Aβ42 aggregates formed with and without drugs. Freshly cleaved, high-quality V1 mica discs were prepared by stripping the top layer with adhesive tape to create a clean, atomically flat surface for sample deposition.

A 20 μL aliquot of each sample was deposited onto the etched mica and incubated for 5 min to allow physical adsorption. After incubation, the mica was rinsed with ultrapure water and air-dried overnight in a fume hood with gentle airflow. AFM measurements were performed in tapping mode using a Multimode 8 system (Bruker) equipped with a Scanasyst-Air probe (triangular cantilever; resonant frequency 70 kHz; spring constant 0.4 N/m; tip radius 2 nm; Bruker). Images were acquired with a scan size of 5 μm × 5 μm at a resolution of 512 lines. Nanoscope Analysis 1.7 software (Bruker) was used for image analysis.

### 3.7 Preparation of Aβ42 Oligomers

AβO purchased from China Peptides (Shanghai, China), were used to induce cytotoxicity in BV2 cells, serving as a model for AβO-induced neurotoxicity. To prepare the AβO, Aβ42 monomers were dissolved in DMSO and diluted to 1 mg/mL with PBS. Oligomerization was achieved by incubating this solution at 37 °C for 24 h; formation was confirmed by transmission electron microscopy. The resulting AβO were lyophilized, shipped at 4 °C, and stored at −80 °C. For cell culture experiments, lyophilized AβO were reconstituted in DMSO to 5 μM, sonicated for 15 min, and further diluted in Dulbecco’s Modified Eagle Medium (DMEM; Invitrogen) to the desired final concentrations.

### 3.8 Cell Culture

The immortalized mouse microglial cell line BV2, obtained from the National Infrastructure of Cell Line Resource (Beijing, China), was used in this study. Cells were cultured in DMEM supplemented with 10% fetal bovine serum (FBS; Gibco) and 1% penicillin–streptomycin (Sigma-Aldrich). Cells were maintained at 37 °C in a humidified atmosphere containing 5% CO2. For optimal growth, the medium was changed regularly, and cells were subcultured at approximately 80% confluence using 0.25% trypsin (Gibco).

### 3.9 Cell Viability Assay

The cytotoxicity of anidulafungin and AβO on BV2 cells was assessed using CCK-8 kits (NCM Biotech). Anidulafungin (50 mM stock solution) was prepared in DMSO and diluted to the desired concentrations in DMEM. BV2 cells (5 × 10^3^ cells/well) were seeded into 96-well plates and incubated overnight. Cells were then treated with anidulafungin (10 nM–5 μM) or AβO (2.5–20 μM) for 24 h. To evaluate the neuroprotective effects of anidulafungin against AβO, BV2 cells were cotreated with 5 μM AβO and varying concentrations of anidulafungin (0–1 μM) for 24 h. After treatments, 10 μL of CCK-8 reagent was added to each well, followed by incubation for 1.5 h at 37 °C. Sample absorbance was measured at 450 nm using a spectrophotometer.

### 3.10 Molecular Docking Simulations

Molecular docking simulations were performed to investigate the potential binding of anidulafungin to various Aβ42 structures, using Autodock Vina within the AutoDock Tools.^60^ Aβ42 structures were obtained from the Protein Data Bank (PDB), including an α-helical monomer (PDB ID: 1Z0Q), a β-sheet monomer isolated from a cryo-EM fibrils structure (PDB ID: 5OQV), Aβ42 tetramers (PDB ID: 6RHY), and “LS”-shaped (PDB ID: 5OQV), ν-shaped (PDB ID: 8EZD), and υ-shaped (PDB ID: 8EZE) fibrils. Anidulafungin’s structure was retrieved from PubChem (CID: 166548) and converted to a 3D PDB format using OpenBabel. AutoDock Tools was used to prepare both the Aβ42 macromolecules and the anidulafungin ligand for docking. Autogrid generated grid box parameters for the Aβ42 monomers (60 × 60 × 60 Å, centered on the molecule), while larger boxes (100 × 100 × 100 Å) were used for tetramer and fibril structures. All other docking parameters were set to default values. Results were analyzed with AutoDock Tools and visualized using Discovery Studio v24.1.0.

### 3.11 Statistical Analysis

Statistical analyses were performed using GraphPad Prism 9. To compare mean cell viability across different BV2 cell treatment groups, one-way ANOVA followed by Tukey’s post hoc test was used for multiple comparisons. When analyzing primary nucleation rate constants, one-way ANOVA followed by Dunnett’s post hoc test was employed to compare the rate constants obtained under different anidulafungin concentrations against the Aβ42 control group. For all analyses, a *P*-value <0.05 was considered statistically significant.
